# Comparative Study on the Cytoprotective Effects of Activated Protein C Treatment in Nonsteatotic and Steatotic Livers under Ischemia-Reperfusion Injury

**DOI:** 10.1155/2015/635041

**Published:** 2015-10-11

**Authors:** Akitoshi Matsuda, Naohisa Kuriyama, Hiroyuki Kato, Akihiro Tanemura, Yasuhiro Murata, Yoshinori Azumi, Masashi Kishiwada, Shugo Mizuno, Masanobu Usui, Hiroyuki Sakurai, Shuji Isaji

**Affiliations:** Department of Hepatobiliary Pancreatic and Transplant Surgery, Mie University Graduate School of Medicine, Edobashi 2-174, Tsu, Mie 514-8507, Japan

## Abstract

Activated protein C (APC) has cytoprotective effects on liver ischemia-reperfusion injury (IRI). However, it is unclear whether APC is beneficial in steatotic liver IRI. We compared the cytoprotective effects of APC in nonsteatotic and steatotic liver IRI.* Methods.* Mice fed either normal diets (ND mice) or high fat diets (HF mice), were treated with APC or saline (control) and were performed 60 min partial IRI. Moreover, primary steatotic hepatocytes were either untreated or treated with APC and then incubated with H_2_O_2_.* Results.* APC significantly reduced serum transaminase levels and the inflammatory cells infiltration compared with control at 4 h in ND mice and at 24 h in HF mice. APC inhibited sinusoidal endothelial injury in ND mice, but not in HF mice. In contrast, APC activated adenosine monophosphate-activated protein kinase (AMPK) phosphorylation in HF mice, but not in ND mice. In the in vitro study, APC significantly increased AMPK phosphorylation, ATP concentration, and survival rates of hepatocytes compared with control.* Conclusion.* During IRI in normal liver, APC attenuated initial damage by inhibiting inflammatory cell infiltration and sinusoidal endothelial injury, but not in steatotic liver. However, in steatotic liver, APC might attenuate late damage via activation of AMPK.

## 1. Introduction

Hepatic steatosis is a major risk factor for liver resection and transplantation. Recently, the epidemic of obesity in developed countries has increased, along with its attendant complications, including metabolic syndrome and hepatic steatosis. Between 6 and 33% of individuals in the general population [1] and 70–80% of obese patients have hepatic steatosis. Liver transplants using steatotic liver grafts are associated with a high primary graft nonfunction rate compared with nonsteatotic livers [2, 3]. Along with transplantation, steatotic livers have a negative impact in other clinical situations, such as hepatectomy, shock, and cardiac arrest, which are all subject to warm hepatic ischemia-reperfusion injury (IRI) [4].

Although it is generally accepted that steatotic livers are particularly vulnerable to hepatic IRI, results from animal experiments indicate that the mechanisms underlying IRI are different in nonsteatotic and steatotic livers [5]. Cellular hypoxia persists in fatty hepatocytes during IRI because the hepatic sinusoidal space is obstructed due to fat droplet accumulation in the cytoplasm of hepatocytes. This causes a reduction in sinusoidal blood flow [6] and a decrease in ATP synthase and increase in reactive oxygen species (ROS) production within the steatotic liver is induced by the increased level of mitochondrial uncoupling protein-2, a mitochondrial inner membrane protein that mediates proton leakage across the inner membrane by uncoupling substrate oxidation from ATP synthesis [7–9]. However, the reason why hepatic IRI is increased in steatotic liver has not yet been fully elucidated.

Among a large number of pharmacological agents to protect against IRI in animal models, activated protein C (APC), an anticoagulant, has been shown to have cytoprotective effects against IRI in several organs [10, 11]. Previously, we reported that APC administration had a cytoprotective effect on hepatic IRI in rat models, by preventing recruitment of inflammatory cells, ameliorating sinusoidal endothelial cell injury, and maintaining sinusoidal blood flow [11]. Previous studies provide evidence supporting the idea of direct cytoprotective actions of APC in which endothelial protein C receptor- (EPCR-) bound APC activates protease-activated receptor 1 (PAR1) to initiate signaling on endothelial cells [12, 13]. In contrast, a recent study using a myocardial IRI model elucidated another mechanism of APC, namely, triggering adenosine monophosphate-activated protein kinase (AMPK) signaling: phosphorylation of AMPK mediates dramatic changes in cell metabolism, cell survival, and other functions [14]. In steatotic liver, mitochondria in hepatocytes produce excessive amounts of reactive oxidative species (ROS) leading to damage of mitochondrial inner membrane proteins and a consequent decrease of mitochondrial adenosine triphosphate (ATP) production [15]. Moreover, hepatic ATP stores are reduced in steatotic livers which are more vulnerable to necrosis after transient hepatic ischemia [16]. To the best of our knowledge, there have been no previous studies investigating whether APC treatment is beneficial against steatotic liver IRI, compared with nonsteatotic liver. On the basis of evidence from these previous studies, we speculate that APC might have a different mechanism in steatotic liver compared with nonsteatotic liver, namely, that APC attenuates I/R injury by preventing the depletion of ATP via AMPK activation, and this effect is specific to steatotic liver.

In the present study, we compared the cytoprotective effects of APC administration between nonsteatotic and steatotic liver IRI in a mouse model, in an attempt to elucidate the theoretical mechanism by which APC attenuates liver damage specifically in steatotic liver.

## 2. Materials and Methods

### 2.1. Animals

**Figure 1:**
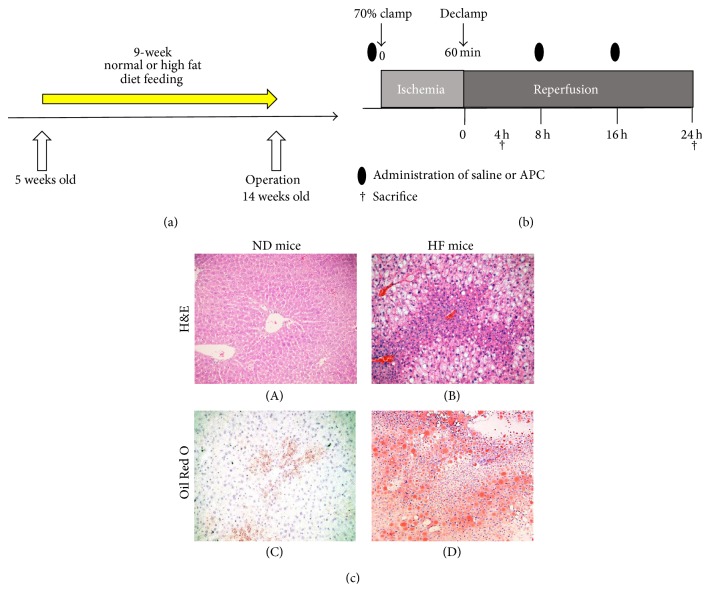
Development of steatotic mice and method of IRI. Five-week-old C57BL/6 male mice were fed either a normal diet (ND mice) or a high fat diet (HF mice) for 9 weeks (a). Blood supply to the cephalad lobes of the liver was interrupted for 60 min using an atraumatic clip. After 60 min of ischemia, the clip was removed. APC or saline solution was administered just prior to surgery and at 8 h and 16 h after reperfusion. Mice were sacrificed 4 h or 24 h after reperfusion (b). Representative H&E ((c)-(A), (c)-(B)) and Oil Red O staining ((c)-(C), (c)-(D)) of liver tissue after 9 weeks on the experimental diets. There was no steatosis in ND mice ((c)-(A), (c)-(C)). After 9 weeks of HF diet feeding, macrosteatosis was observed using H&E staining and Oil Red O staining ((c)-(B), (c)-(D)). The original magnification was ×100.

Five-week-old male mice (C57BL/6; Japan SLC, Inc.) were fed either a normal diet (ND mice) or a high fat diet (60% calories from fat; Research Diets number D12492) (HF mice) for 9 weeks (Figure 1(a)). All experiments were conducted in compliance with the Guideline for Animal Experiments in Mie University Graduate School of Medicine.

### 2.2. Activated Protein C

Human plasma-derived APC was kindly provided by the Chemo-Sero-Therapeutic Research Institute (Kumamoto, Japan). ND and HF mice were randomly assigned to either APC treatment or control groups, resulting in the following four groups being established: (1) ND-APC, (2) ND-Control, (3) HF-APC, and (4) HF-Control. The number of animals used in each group was 12. APC (0.2 mg/kg of body weight) or saline solution (the volume equivalent to APC solution) was intravenously administered just prior to surgery and at 8 h and 16 h after reperfusion [11, 14].

### 2.3. Model of Partial Lobar Liver IRI

A warm hepatic IRI model was established in 14-week-old male mice (i.e., after 9 weeks of diet feeding). Mice were anaesthetized with isoflurane and livers were exposed through a midline laparotomy. The arterial and portal venous blood supplies were interrupted to the cephalad lobes of the liver for 60 min using an atraumatic clip. The right hepatic lobe and the caudate lobe were perfused to prevent intestinal congestion. After 60 min of ischemia, the clip was removed, thus initiating hepatic reperfusion. Mice were sacrificed 4 h or 24 h after reperfusion (*n* = 6 in each group; Figure 1(b)). Body weight was measured before the operation.

### 2.4. Serum Transaminases

Serum alanine transaminase (ALT) and serum aspartate transaminase (AST) levels were measured using a commercially available kit (Wako Pure Chemical Industries Ltd., Osaka, Japan), following manufacturer's instructions.

### 2.5. Histology and Immunohistochemistry

Liver histology and immunohistochemistry were performed as previously reported [17, 18]. Liver specimens embedded in paraffin were processed for hematoxylin and eosin (H&E) staining. The histological damage due to hepatic IRI was assessed using the modified Suzuki score [19], as well as by the extent of the necrotic area. In the modified Suzuki score, sinusoidal congestion, hepatocyte necrosis, and ballooning degeneration were graded from 0 to 4. No necrosis, congestion, or centrilobular ballooning was given a score of 0, whereas severe congestion and ballooning degeneration and 60% lobular necrosis were given a score of 4. The degree of hepatic necrosis 24 h after IRI was assessed in H&E-stained paraffin sections; H&E stains were digitally photographed and the percent of necrosis was quantified using NIH Image J software in a manner blinded to the different experimental groups, as previously described [20]. Liver steatosis was evaluated using Oil Red O staining. Immunohistochemistry was performed using Ly6G (1A8) from BioLegend (San Diego, CA), MAC-1 (M1/70) and PECAM-1 (MEC13.3) from BD Biosciences (San Jose, CA), and phospho-AMPK (p-AMPK) (40H9) from Cell Signaling Technology (Beverly, MA), with all antibodies used at optimal dilutions. The results were evaluated by an average of 10 times' counting in 40 high-power (×400 magnification) fields per section.

### 2.6. Western Blot Analysis

Western blots were performed as described previously [17]. PVDF membranes were incubated with antibodies against PECAM-1 (epitope within extracellular domain; SC-28188; Santa Cruz Biotechnology, Santa Cruz, CA) and p-AMPK (40H9; Cell Signaling Technology). After development, membranes were stripped and reblotted with antibodies against AMPK (D5A2; Cell Signaling Technology) and actin (Cell Signaling Technology). Prestained molecular weight markers (Protein MultiColor III; BioDynamics Laboratory Inc., Tokyo, Japan) served as standards. Relative quantities of protein were determined using a densitometer (NIH Image J software).

### 2.7. Cell Culture

Primary steatotic hepatocytes were isolated from HF mice. To isolate primary murine hepatocytes, anesthetized mice were subjected to a midline laparotomy and cannulation of the portal vein followed by liver perfusion with an EGTA-chelating perfusion buffer (EGTA: 190 mg, glucose: 900 mg, HEPES: 10 mL of 1 M stock solution, KCL: 400 mg, Na_2_HPO_4_-2H_2_O: 151 mg, NaCl: 8 g, NaH_2_PO_4_-H_2_O: 7 mg, and NaHCO_3_: 350 mg, made up to 1 L with dH_2_O). After perfusion with 0.4% collagenase buffer (CaCl_2_-2H_2_O: 560 mg, HEPES: 10 mL of 1 M stock solution, KCL: 400 mg, Na_2_HPO_4_-2H_2_O: 151 mg, NaCl: 8 g, NaH_2_PO_4_-H_2_O: 7 mg, NaHCO_3_: 350 mg, and collagenase P: 400 mg, made up to 1 L with dH_2_O), livers were minced and cells dispersed in culture medium; hepatocyte and nonparenchymal cells were separated using low-speed centrifugation methods. Isolated mouse steatotic hepatocytes (2 × 10^5^/well) were cultured in DMEM with 10% FBS on 24-well collagen-coated plate at 37°C with 5% CO_2_ for 12 h. Hepatocytes were incubated in the presence or absence of H_2_O_2_ (500 nM) and/or APC (300 nM) and/or compound C (10 *μ*M) (Tocris Bioscience, Bristol, UK), which is an inhibitor of AMPK. After 24 h culture, the cell lysates were prepared for protein evaluation, and the supernatants were collected for cytotoxicity assays. Cell viability was assessed by counting an aliquot in the presence of 0.4% Trypan blue. Cell cytotoxicity was assessed by AST levels in culture media. ATP levels of hepatocytes were measured using a commercially available kit (BioVision, Palo Alto, CA), according to the manufacturer's instructions.

### 2.8. Data Analysis

The results of continuous variables are expressed as the mean value ± standard deviation. Statistical comparisons between groups of normally distributed data were performed using the Mann-Whitney *U* test with SPSS software (SPSS Inc., Chicago, IL). *P* values less than 0.05 were considered statistically significant.

## 3. Results

### 3.1. HF Mice Develop Macrosteatosis

There was no observation of steatosis in ND mice (Figures 1(c)-(A) and 1(c)-(C)). HF mice developed fatty livers which resembled those of human obesity [21]. In our experimental setting, HF mice were characterized by 50% liver steatosis, with macrovesicular fatty infiltration, as assessed by H&E (Figure 1(c)-(B)) and Oil Red O staining (Figure 1(c)-(D)). Moreover, the body weight of 9-week HF mice was significantly higher than that of ND mice (41.3 ± 2.1 versus 27.7 ± 1.5 g, *P* < 0.05).

### 3.2. APC Ameliorates Hepatocellular Injury in ND and HF Mice at Different Time Points

**Figure 2:**
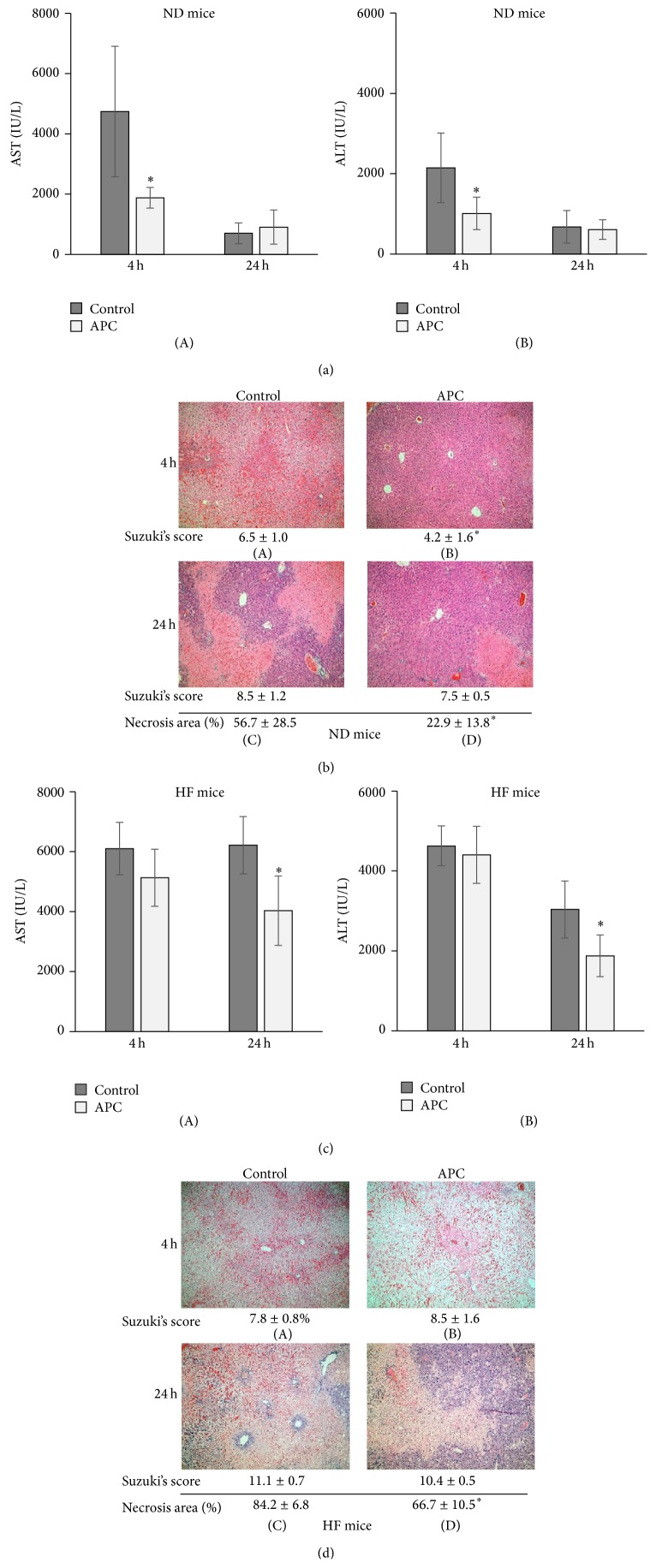
Transaminase levels and histology in ND and HF mice. Serum AST and ALT levels at 4 h were significantly decreased in ND-APC compared with ND-Control mice (^∗^ *P* < 0.05). There was no significant difference at 24 h between ND-APC and ND-Control mice ((a)-(A), (a)-(B)). H&E staining showed that the ND-APC had significantly preserved lobular architecture and reduced intrasinusoidal/vascular congestion compared with ND-Control at 4 h ((b)-(A), (b)-(B)). The necrotic area within livers was significantly reduced in ND-APC compared with ND-Control mice at 24 h (^∗^ *P* < 0.05) ((b)-(C), (b)-(D)). While serum AST and ALT levels at 24 h were significantly decreased in HF-APC compared with HF-Control mice (^∗^ *P* < 0.05), there was no significant difference at 4 h between HF-APC and HF-Control mice ((c)-(A), (c)-(B)). In HF mice, however, liver tissues in both the HF-APC and HF-Control groups showed marked changes in vacuolization and intrasinusoidal/vascular congestion ((d)-(A), (d)-(B)). Although there was more severe necrosis in HF mice than in ND mice, the necrotic area of hepatocytes was significantly reduced in HF-APC compared with HF-Control mice at 24 h (^∗^ *P* < 0.05) ((d)-(C), (d)-(D)). The numbers under the pictures show the modified Suzuki score and the percentage of necrotic area (%). The original magnification was ×100 (b, d).

In ND mice, APC treatment significantly reduced serum AST and ALT levels at 4 h (AST: 1,879 ± 344 versus 4,741 ± 2,167 IU/L; ALT: 1,012 ± 403 versus 2,146 ± 866 IU/L, *P* < 0.05). However, there were no significant differences at 24 h between ND-APC and ND-Control mice (AST: 903 ± 565 versus 700 ± 344 IU/L; ALT: 610 ± 243 versus 678 ± 403 IU/L) (Figures 2(a)-(A) and 2(a)-(B)). In the assessment of histological damage, necrotic area could not be accurately assessed at 4 h because development of necrosis was scarce. The modified Suzuki score at 4 h was significantly lower in ND-APC than in ND-Control mice: 4.2 ± 1.6 versus 6.5 ± 1.0, *P* < 0.05 (Figures 2(b)-(A) and 2(b)-(B)), while at 24 h there was no significant difference between the two groups: 7.5 ± 0.5 versus 8.5 ± 1.2 (Figures 2(b)-(C) and 2(b)-(D)). The necrotic area at 24 h was significantly lower in ND-APC than in ND-Control mice: 22.9 ± 13.8 versus 56.7 ± 28.5%, *P* < 0.05 (Figures 2(b)-(C) and 2(b)-(D)).

In HF mice, APC treatment did not significantly reduce serum AST or ALT levels at 4 h compared with HF-Control mice (AST: 5,130 ± 954 versus 6,103 ± 873 IU/L; ALT: 4,403 ± 715 versus 4,627 ± 499 IU/L), while it significantly improved steatotic liver function at 24 h (AST: 4,032 ± 1,160 versus 6,218 ± 954 IU/L; ALT: 1,876 ± 523 versus 3,037 ± 715 IU/L, *P* < 0.05) (Figures 2(c)-(A) and 2(c)-(B)). In the assessment of histological damage, the modified Suzuki score was very difficult to assess in steatotic liver due to the large number of fatty droplets, and necrotic area could not be evaluated at 4 h because development of necrosis was scarce. The modified Suzuki scores at 4 h and 24 h were not significantly different between HF-APC and HF-Control mice: 8.5 ± 1.6 versus 7.8 ± 0.8 at 4 h (Figures 2(d)-(A) and 2(d)-(B)); 10.4 ± 0.5 versus 11.1 ± 0.7 at 24 h (Figures 2(d)-(C) and 2(d)-(D)). In contrast, the necrotic area at 24 h was significantly lower in HF-APC than in HF-Control mice: 66.7 ± 10.5 versus 84.2 ± 6.8% (*P* < 0.05) (Figures 2(d)-(C) and 2(d)-(D)).

### 3.3. APC Prevents Intrahepatic Leucocyte Infiltration in ND and HF Mice

**Figure 3:**
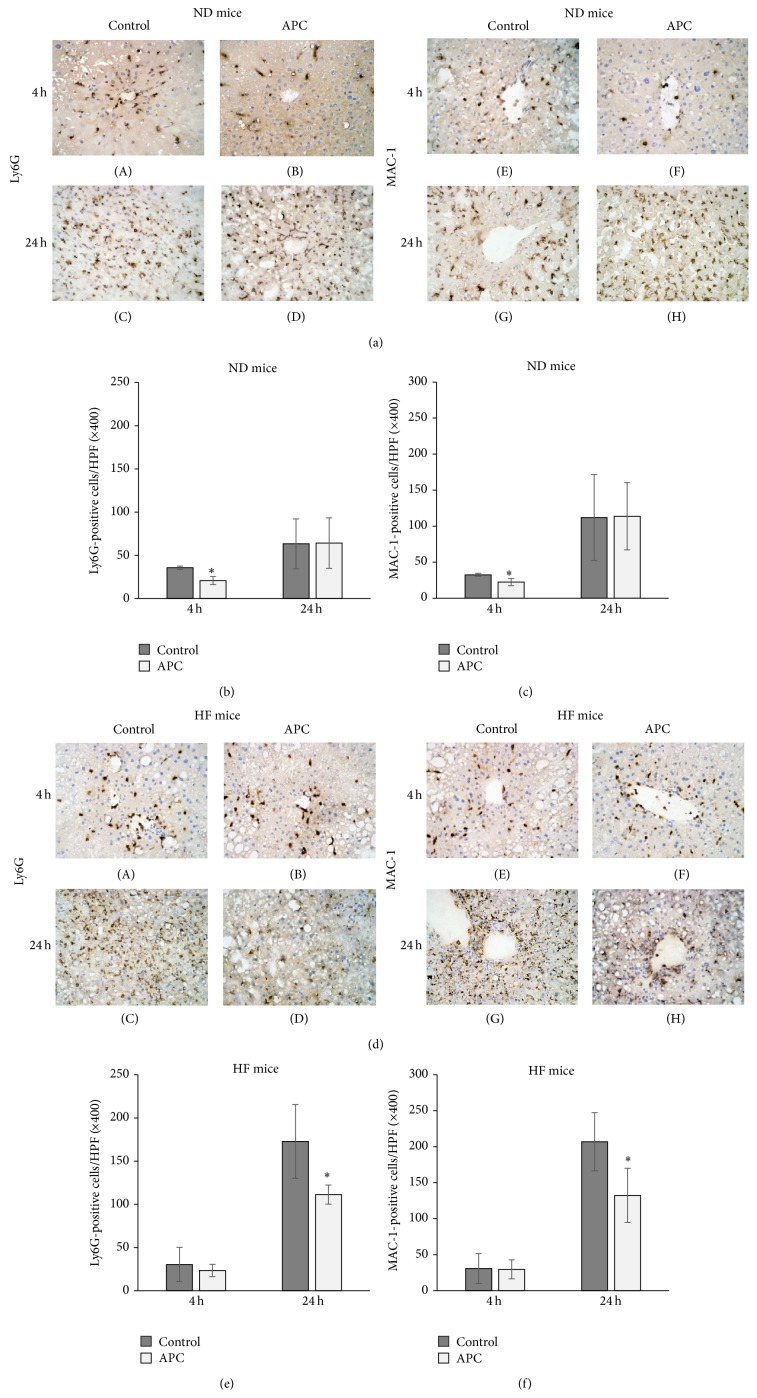
Leukocyte infiltration 4 h and 24 h after reperfusion. Infiltration of Ly6G-positive cells ((a)-(A), (a)-(B), and (b)) and MAC-1-positive cells ((a)-(E), (a)-(F), and (c)) in the liver was significantly decreased in ND-APC compared with ND-Control mice at 4 h (^∗^ *P* < 0.05). However, in HF mice, infiltration of Ly6G-positive cells ((d)-(C), (d)-(D), and (e)) and MAC-1-positive cells ((d)-(G), (d)-(H), and (f)) was significantly decreased in HF-APC livers compared with HF-Control livers at 24 h (^∗^ *P* < 0.05). There was no significant difference at 4 h between HF-APC and HF-Control mice. The original magnification was ×400 (a, d).

To determine whether APC affects local leucocyte infiltration, we assessed Ly6G-positive cells and MAC-1-positive cells using immunohistochemical staining. The number of Ly6G-positive cells in the liver was significantly decreased in ND-APC mice compared with ND-Control mice at 4 h (20.9 ± 4.6 versus 35.7 ± 1.9, *P* < 0.05) (Figures 3(a)-(A), 3(a)-(B), and 3(b)). However, at 24 h, there were no significant differences in the number of Ly6G-positive cells that had infiltrated when comparing the two groups (64.1 ± 29.2 versus 63.4 ± 28.8) (Figures 3(a)-(C), 3(a)-(D), and 3(b)). The number of MAC-1-positive cells in the liver was significantly decreased in ND-APC compared with ND-Control mice at 4 h (22.3 ± 4.9 versus 32.5 ± 1.9, *P* < 0.05) (Figures 3(a)-(E), 3(a)-(F), and 3(c)). However, at 24 h, there were no significant differences in the number of MAC-1-positive cells between the two groups (113.8 ± 46.7 versus 111.9 ± 59.6) (Figures 3(a)-(G), 3(a)-(H), and 3(c)).

In HF mice, there were no significant differences in the number of Ly6G-positive cells at 4 h between the two groups (23.4 ± 7.1 versus 30.4 ± 19.9) (Figures 3(d)-(A), 3(d)-(B), and 3(e)), while at 24 h the number was significantly decreased in HF-APC mice compared with HF-Control mice (111.4 ± 11.0 versus 172.8 ± 42.6, *P* < 0.05) (Figures 3(d)-(C), 3(d)-(D), and 3(e)). Although there were no significant differences in the number of MAC-1-positive cells at 4 h between the two groups (29.5 ± 13.2 versus 30.7 ± 20.8) (Figures 3(d)-(E), 3(d)-(F), and 3(f)), their number was significantly decreased in HF-APC mice compared with HF-Control mice at 24 h (132.4 ± 37.7 versus 206.8 ± 40.4, *P* < 0.05) (Figures 3(d)-(G), 3(d)-(H), and 3(f)).

### 3.4. APC Prevents Sinusoidal Endothelial Cell Damage in ND Liver, but Not in HF Liver

**Figure 4:**
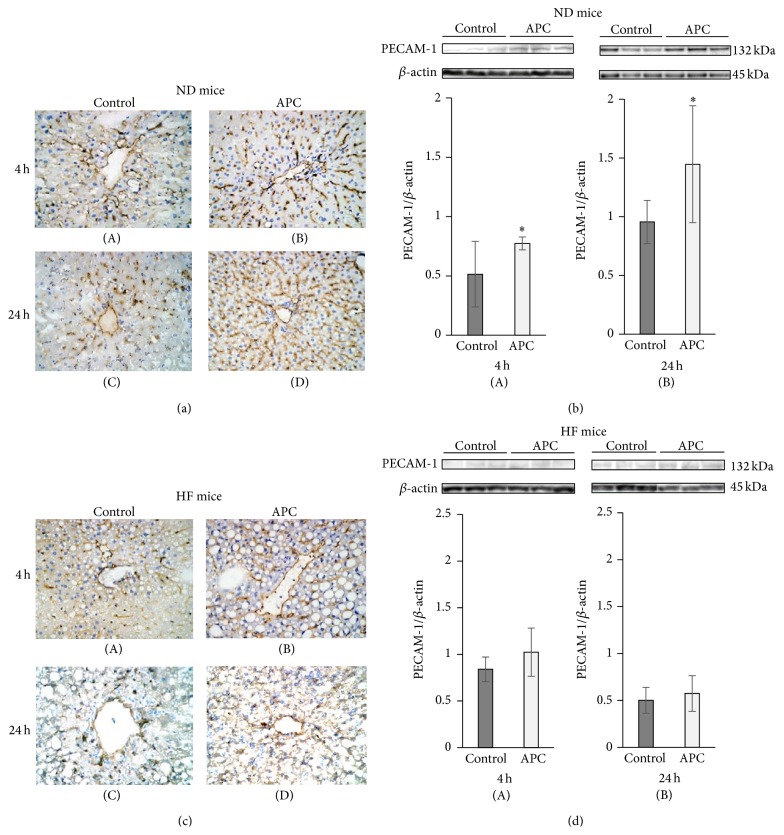
Sinusoidal endothelial cell damage in ND and HF mice. IRI disrupted sinusoidal vasculature regardless of APC administration in HF mice. In the liver tissue of ND mice, there was significant large number of sinusoidal endothelial cells, which were stained with PECAM-1 antibody in ND-APC than in ND-Control mice at 4 h ((a)-(A), (a)-(B), and (b)-(A)) and 24 h ((a)-(C), (a)-(D), and (b)-(B)). In liver tissue of HF mice, sinusoidal endothelial cells which were stained with PECAM-1 antibody were disrupted regardless of APC administration at both 4 h ((c)-(A), (c)-(B), and (d)-(A)) and 24 h ((c)-(C), (c)-(D), and (d)-(B)).

PECAM-1 expression is readily detected on the intact vascular endothelium of naïve livers without IRI [18]. However, while PECAM-1 expression was relatively preserved on the vascular endothelium of ND-APC livers after IRI, it was largely absent from the vasculature of control livers, particularly at 4 h and 24 h (Figures 4(a)-(A)–4(a)-(D)). Indeed, the full length PECAM-1 (132 kDa) was detected in ND-APC livers and markedly depressed in ND-Control livers at 4 h and 24 h (4 h: 0.77 ± 0.06 versus 0.51 ± 0.28; 24 h: 1.45 ± 0.50 versus 0.96 ± 0.18, *P* < 0.05) (Figures 4(b)-(A) and 4(b)-(B)). In contrast, sinusoidal endothelial structures were severely disrupted regardless of the APC administration at 4 h and 24 h in HF mice (Figures 4(c)-(A)–4(c)-(D)). There were no significant differences in relative quantities of PECAM-1 between HF-APC and HF-Control livers (4 h: 1.02 ± 0.26 versus 0.84 ± 0.13; 24 h: 0.57 ± 0.19 versus 0.50 ± 0.14) (Figures 4(d)-(A) and 4(d)-(B)). Taken together, these data suggest that APC reduced sinusoidal damage due to hepatic IRI in ND livers, which is cytoprotective effect of APC, but this effect was abolished in steatotic livers, meaning that the other effects of APC needed to be considered, except in the case of endothelial cells.

### 3.5. APC Administration Activates AMPK Phosphorylation at 4 h in HF Livers, but Not in ND Livers

**Figure 5:**
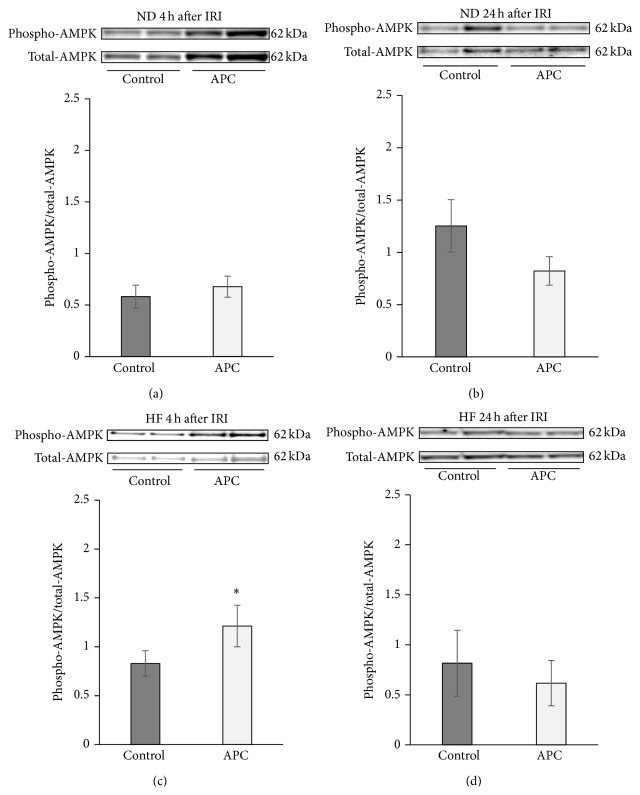
Phosphorylation of AMPK in steatotic liver tissue. In ND mice, there was no significant difference in phosphorylation of AMPK regardless of the APC administration at 4 or 24 h (a, b). APC significantly increased phosphorylation of AMPK at 4 h in HF mice (^∗^ *P* < 0.05) (c). At 24 h, however, there was no significant difference in phosphorylation of AMPK between HF-APC and HF-Control mice (d).

**Figure 6:**
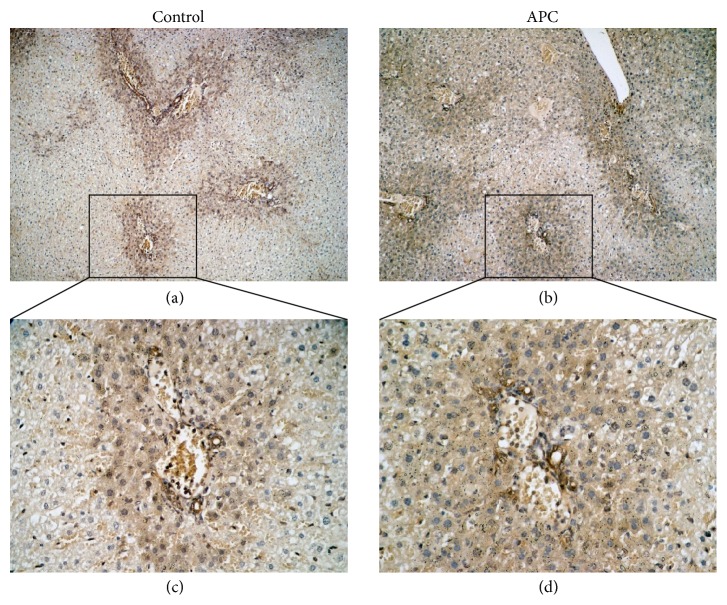
Immunohistochemical staining of phospho-AMPK in steatotic liver. Phospho-AMPK was predominantly expressed by the surviving steatotic hepatocytes particularly around the portal triad. The original magnification was ×100 (a, c) or ×400 (b, d).

According to previous reports, APC is thought to potentiate the phosphorylation of AMPK, a serine-threonine kinase which maintains cellular energy stores and prevents energy depletion [14, 22, 23]. When we focused on the phosphorylation of AMPK in ND mouse livers, there were no significant differences at 4 h or 24 h between ND-APC and ND-Control mice (4 h: 0.58 ± 0.11 versus 0.67 ± 0.10; 24 h: 1.45 ± 0.47 versus 1.09 ± 0.34) (Figures 5(a) and 5(b)). In contrast, AMPK phosphorylation was significantly increased in HF-APC mice compared with HF-Control mice at 4 h (1.21 ± 0.21 versus 0.83 ± 0.13, *P* < 0.05) (Figure 5(c)). However, at 24 h, there were no significant differences between the two groups in relative quantities of AMPK phosphorylation (0.82 ± 0.33 versus 0.62 ± 0.22) (Figure 5(d)). We then focused on the localization of p-AMPK in HF livers. Immunohistochemical staining of the liver revealed that p-AMPK was predominantly expressed by the steatotic hepatocytes in the periportal area (zone I) 4 h after reperfusion (Figure 6).

### 3.6. APC Improves Hepatocyte Survival via Upregulation of AMPK Phosphorylation In Vitro

According to the data from the in vivo AMPK analysis and evidence that p-AMPK was mainly detected in surviving hepatocytes at 4 h after IRI, we isolated primary steatotic hepatocytes, which are considered the main site of energy storage in steatotic liver, and evaluated the levels of AMPK phosphorylation and the degree of energy depletion in the presence or absence of APC administration.

**Figure 7:**
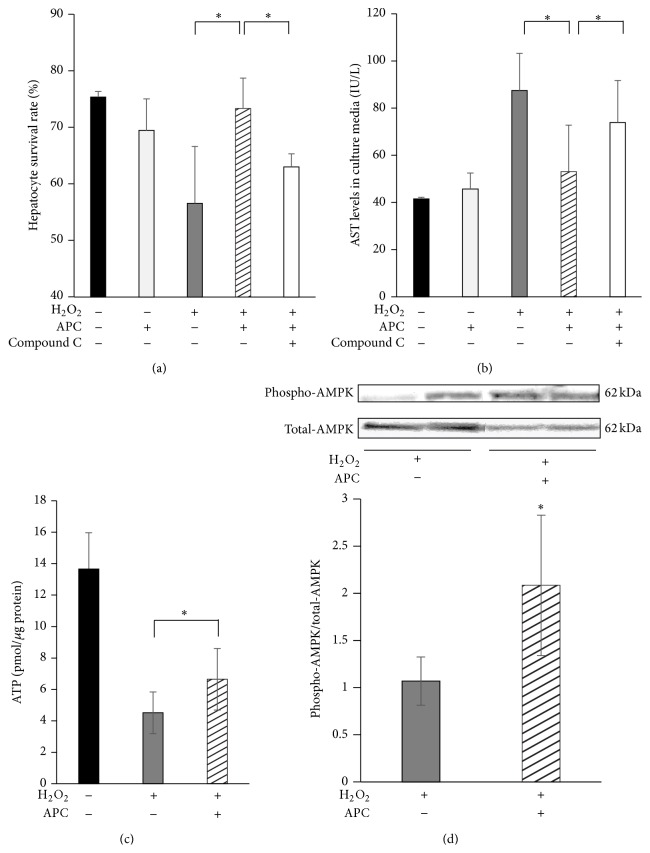
Direct effects of APC on steatotic hepatocytes in vitro. In cells treated with H_2_O_2_ + APC, the survival rates of steatotic hepatocytes were significantly increased compared with those of cells treated with H_2_O_2_ alone (^∗^ *P* < 0.05). By adding an inhibitor of AMPK (compound C), the survival rate was significantly decreased (^∗^ *P* < 0.05) (a). Cell cytotoxicity, which was calculated by the AST level in the culture media, was significantly decreased in the H_2_O_2_ + APC treatment, compared with H_2_O_2_ treatment alone. By adding compound C, the AST levels were significantly increased (b). APC increased the ATP concentration in hepatocytes which were cultured for 24 h with H_2_O_2_ (c). Representative western blots for phospho-AMPK (p-AMPK) and total-AMPK (t-AMPK) in hepatocytes which were cultured for 24 h with H_2_O_2_ are shown; APC increased AMPK phosphorylation (^∗^ *P* < 0.05) (d).

The survival rates of steatotic hepatocytes were 75.4 ± 1.0% in the sham group and 69.5 ± 5.6% in response to APC alone, while they were only 56.6 ± 10.1% in response to H_2_O_2_ alone. In response to H_2_O_2_ + APC, they were significantly increased (to 73.3 ± 5.4%) compared with H_2_O_2_ treatment alone. By adding an inhibitor of AMPK (compound C), the survival rate was significantly decreased to 63.0 ± 2.3% (Figure 7(a)). AST levels in the culture medium, an index of cell cytotoxicity, were 41.5 ± 0.7 IU/L in the sham group and 45.7 ± 6.8 IU/L in response to APC alone, while they were increased to 87.5 ± 15.7 IU/L in response to H_2_O_2_ alone. In the H_2_O_2_ + APC treatment, they were significantly decreased (to 53.1 ± 19.7 IU/L) compared with H_2_O_2_ treatment alone. By adding compound C, the AST levels were significantly increased (to 73.9 ± 17.9 IU/L; Figure 7(b)). ATP levels of steatotic hepatocytes, after 24 h incubation with H_2_O_2_, were significantly preserved in the presence of APC, compared with the respective controls (6.64 ± 1.96 versus 4.51 ± 1.32 pmol/*μ*g protein, *P* < 0.05; Figure 7(c)). The relative quantities of AMPK phosphorylation in steatotic hepatocytes (as assessed by western blot analysis) were significantly higher in the presence of APC compared with controls (2.08 ± 0.75 versus 1.07 ± 0.26, *P* < 0.01; Figure 7(d)).

## 4. Discussion

In the present study, both nonsteatotic and steatotic mice treated with APC showed significant improvements with respect to serum transaminase levels, liver histological damage, and leukocyte recruitment, and these effects were mainly observed at 4 h in nonsteatotic livers and at 24 h in steatotic livers. In nonsteatotic liver, the transaminase levels which were markedly increased at 4 h could be attenuated by APC treatment, while these levels were significantly decreased at 24 h in both groups, showing no significant difference. The modified Suzuki score of liver damage, which was derived from a combination of congestion, centrilobular ballooning, and necrosis, was significantly reduced by APC treatment at 4 h, but not at 24 h. The level of inflammatory cell infiltration assessed by immunochemical staining of Ly6G and MAC-1 was significantly attenuated at 4 h by APC treatment, while at 24 h it was significantly increased, showing no significant difference between the two groups. In contrast, necrotic area was significantly attenuated at 24 h by APC treatment. Taken together, these results suggest that APC treatment for IRI in nonsteatotic (normal) liver attenuated initial liver damage by inhibiting inflammatory cell infiltration, which in turn significantly reduces necrosis at 24 h.

In contrast, in steatotic liver, APC treatment did not attenuate transaminase levels at 4 h, which were markedly increased and significantly higher than those in nonsteatotic liver, although it did attenuate the levels at 24 h. Histological assessment showed that APC treatment reduced necrotic area at 24 h, although it did not affect the modified Suzuki score at 4 h or 24 h. The number of inflammatory cells infiltrating, which was not significantly reduced at 4 h by APC treatment, was significantly increased at 24 h in both groups but this effect was significantly attenuated by APC treatment. These results show that APC treatment for IRI in steatotic liver does not affect initial liver damage, but it may attenuate late damage, suggesting that APC might act through a pathway in addition to an anti-inflammatory cytoprotective effect. In previous reports, the presence of macrosteatosis significantly increased the incidence in primary graft dysfunction [24, 25] and decreased patient survival after orthotopic liver transplantation. Moreover, livers with macrovesicular fat deposition have lower tolerance to IRI compared with microsteatotic livers [26, 27]. Our steatotic mice had significantly increased IRI, shown by higher transaminase levels and disrupted liver histology compared with nonsteatotic liver, although serum aminotransferase levels and liver histological outcomes were improved by APC treatment in both nonsteatotic and steatotic mice. Particularly when we focused on the time point of 24 h, post-IRI livers of the steatotic mice showed prolonged parenchymal injury, compared with nonsteatotic mice whose liver injury was already settled down from IRI.

APC treatment in nonsteatotic liver prevented sinusoidal endothelial injury at 4 h and at 24 h, as shown by the expression of PECAM-1 (which is a major constituent of the endothelial cell intercellular junctions and a negative regulator of inflammatory responses [28, 29]). However, APC treatment in steatotic liver did not show any effect on injury until 24 h. APC provides a direct cytoprotective effect on sinusoidal endothelial cells through PAR1 signaling via EPCR-bound APC activation [11]. When we compared the structure of endothelial cells positively stained by PECAM-1 between nonsteatotic and steatotic livers (as shown in Figures 4(a) and 4(c)), sinusoidal endothelial structures were severely disrupted regardless of the APC treatment in steatotic liver, while these structures in nonsteatotic liver were well preserved even in mice without APC treatment. Consequently, we propose that APC treatment could not exert a cytoprotective effect on endothelial vasculature in steatotic liver because it was too severely damaged.

Recently, Wang et al. demonstrated that APC strongly attenuated acute myocardial injury by activating AMPK, an effect that was largely independent of its anticoagulant function. This was shown in both in vivo and ex vivo mouse model systems: cardiomyocytes expressed EPCR and APC directly triggered AMPK phosphorylation in cardiomyocytes by enhancing the Ca2+/CaMKK*β* activity by EPCR- and PAR1-dependent mechanisms [14]. AMPK activates ATP-generating pathways and downregulates ATP-consuming anabolic pathways. We therefore focused on AMPK phosphorylation as a pathway additional to the anti-inflammatory cytoprotective effect of APC. In the present study, APC administration in steatotic liver enhanced its phosphorylation at 4 h, but not at 24 h. In contrast, in nonsteatotic liver, APC administration did not influence AMPK phosphorylation. Histologically, APC treatment did not attenuate liver damage at 4 h, while it significantly reduced the percentage of liver necrosis at 24 h. According to these results, we consider that APC might prevent ATP depletion at 4 h via activation of AMPK phosphorylation, which in turn might partly attenuate the necrosis at 24 h in I/R injury. In our in vitro study, which included a specific inhibitor of AMPK (compound C), the survival rates of steatotic hepatocytes were significantly increased when APC was added in the culture media, while steatotic hepatocyte survival rates were significantly decreased when compound C was added in the culture media containing APC. Nevertheless, in the in vivo study, APC administration significantly attenuated serum transaminase levels, liver histological damage, and leukocyte recruitment in nonsteatotic liver at 4 h and in steatotic liver at 24 h. Therefore, we propose that the main pathway through which APC exerts its cytoprotective effect is the suppression of neutrophil recruitment, although we believe that APC might also exert cytoprotective effects via activation (phosphorylation) of AMPK in steatotic liver.

Target receptors of APC that might directly act on hepatocytes are still unknown. There have been several reports demonstrating that there are receptors of APC other than EPCR, such as the sphingosine-1-phosphate receptor 1 in intestinal epithelial cells [30] and lung epithelial cells [31], several integrins in macrophages [32], PAR3 in neural cells [33], and the apolipoprotein E receptor 2 in platelets [34]. Further study is required to elucidate which of these specific receptors could be the targets of APC in steatotic hepatocytes.

In conclusion, during IRI in normal liver, APC treatment attenuated initial liver damage by inhibiting inflammatory cells infiltration and sinusoidal endothelial injury, while under IRI in steatotic liver APC did not affect initial liver damage but attenuated late damage, suggesting the existence of a pathway additional to the anti-inflammatory cytoprotective effect of APC which may be via activation of AMPK.
